# An unusual complication of polyarteritis nodosa with massive retroperitoneal hemorrhage: a case report

**DOI:** 10.1186/1755-7682-3-31

**Published:** 2010-11-11

**Authors:** Prashanth Peddi, Jagadeesh K Kalavakunta, Madhavi Annakula, John R Armstrong

**Affiliations:** 1 Department of Internal Medicine, Michigan State University, Lansing, MI, USA; 2 Department of Internal Medicine, Michigan State University, Kalamazoo Center for Medical studies, Kalamazoo, MI, USA; 3 Department of Critical Care Medicine, Sparrow Hospital, East Lansing, MI, USA

## Abstract

**Background and Case report:**

Polyarteritis Nodosa (PAN) is a systemic necrotizing vasculitis that affects medium-sized and occasionally involves small arteries leading to the disruption of the internal and external elastic lamina and contribute to the development of aneurysms. Aneurysms develop at bifurcation of major blood vessels; they are prone to thrombosis, rupture and haemorrhage. Retroperitoneal haemorrhage around kidneys was previously reported in patients with PAN. We report a case of massive retroperitoneal bleeding from inferior pancreaticoduodenal artery aneurysm rupture in a 70-year-old female with PAN.

**Conclusion:**

Prognosis of untreated PAN is very poor with 20% 5 year survival rate, therefore early recognition of the disease will prevent catastrophic complications and improves survival.

## Case presentation

A 78-year-old woman presented with fever, anorexia and significant (34 pound) weight loss of three months duration. Three weeks prior to the presentation, she noticed pain and bluish discoloration of her finger tips, which later turned to black with worsening pain. Her past history was significant for hypertension and 35 pack years of smoking. She was febrile with temperature of 100.4°F and other vital signs were stable.

**Figure 1:**
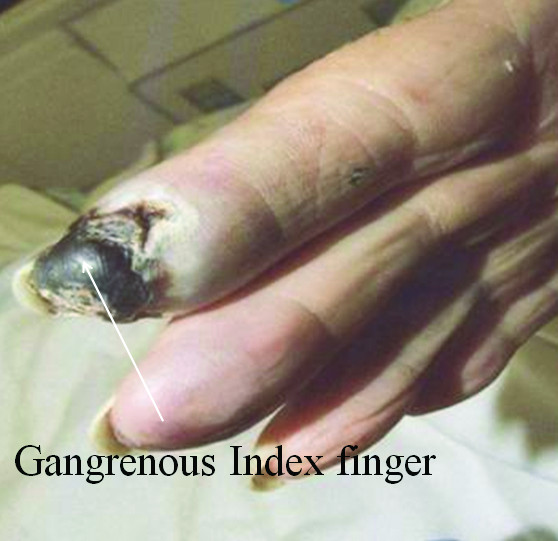
**White arrow pointing the gangrenous index finger**.

**Figure 2:**
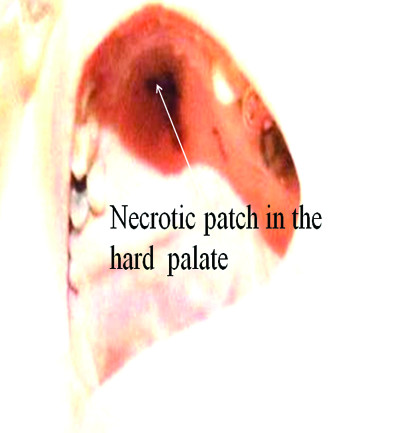
**White arrow pointing black colored lesion in the hard palate**.

Physical examination demonstrated blackish discoloration of her finger tips involving right index, right and left middle finger [Figure 1]. She also had black lesion on her hard palate [Figure 2]. All peripheral pulses were felt symmetrically. Rest of the examination was unremarkable. Her white cell count was elevated at 13,000/mm^3 ^and erythrocyte sedimentation rate was also elevated at 66 mm/hour. Three sets of blood cultures were drawn and empirical antibiotic therapy was begun with piperacillin tazobactum and vancomycin for possible septic embolism.

Workup for endocarditis with transesophageal echocardiogram showed no vegetations. Serologic evaluation for vasculitis with Anti nuclear antibody (ANA), C and P-Anti neutrophil cytoplasmic antibody (ANCA), Anticardiolipin antibody, Rheumatoid factor, and Hepatitis panel did not reveal any abnormality. All three sets of blood cultures showed no growth.

**Figure 3:**
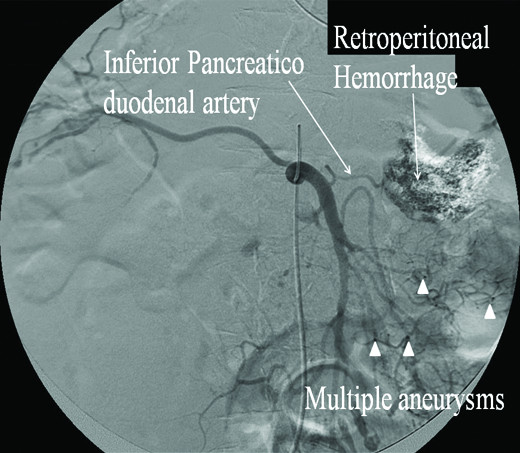
**Mesenteric angiogram showing multiple visceral artery aneurysms (arrow heads) and Inferior pancreaticoduodenal artery along with the retro peritoneal hemorrhage**.

On 3^rd ^day of hospitalization, she developed severe pain and distension of the left side of the abdomen; in association with hypotension and tachycardia. Emergent computer tomography (CT) scan of abdomen showed large retroperitoneal hemorrhage. Further evaluation with mesenteric angiogram revealed multiple visceral artery aneurysms and bleeding from inferior pancreatic duodenal artery aneurysm that was successfully embolized [Figure 3]. She received three units packed red blood cells (PRBC) transfusion and was stabilized hemodynamically.

She was diagnosed with Polyarteritis Nodosa (PAN) after her mesenteric artery aneurysms in accordance with American College of Rheumatology **(**ACR) 1990 criteria and was treated with cyclophosphamide and prednisone with resolution of fever and symptomatic improvement. She subsequently underwent amputation of her gangrenous fingertips and discharged.

## Discussion

Polyarteritis Nodosa (PAN) is a rare form of vasculitis, a multisystem disease, most commonly affects kidney and skin. Prevalence varies from 3-30/100000 [1]. Incidence increases with age; most commonly affects the people in sixth decade as with our patient [2]. The prognosis of untreated PAN is almost always fatal and most patients would die in 2 years.

As with most other patients of PAN our patient had constitutional symptoms like low grade fever, anorexia and weight loss. Hypertension, mild renal insufficiency is commonly seen; however renal insufficiency was not seen in our patient until she developed hemorrhagic shock, which later improved with resuscitation.

In general cutaneous manifestations account for 25-60% which includes livedoreticularis, tender subcutaneous nodules, nonhealing ulcers and gangrene, as seen in our patient [3]. Involvement of abdomen is seen in 50% of people with PAN; symptoms are related to the mesenteric vasculitis causing postprandial abdominal pain, gastrointestinal bleeding, acalculous cholecystitis and pancreatitis. Our patient did not have these symptoms [4].

Aneurysms develop at bifurcation of major blood vessels; they are prone to thrombosis, rupture and hemorrhage, and aneurysms correlates with the severity of the illness [5]. Visceral artery aneurysms rupture results in bleeding into major organs like liver, kidney have been described in the literature [6]. Our patient developed abdominal pain and distension as a result of retroperitoneal hemorrhage from the inferior pancreatic duodenal artery aneurysm rupture, which is unique and has not been reported in the literature to the best of our knowledge. She underwent mesenteric-angiogram which revealed multiple aneurysms involving the superior and inferior pancreatico duodenal arteries and other mesenteric vessels.

Diagnosis of PAN should be confirmed with biopsy whenever possible; however in the absence of biopsy diagnosis can be made with angiogram in with other clinical features in accordance with ACR 1990 criteria to diagnose PAN, which has sensitivity and specificity of 82% and 85% respectively [7]. Workup for secondary causes of vasculitis was negative. Combination of steroids and cyclophosphamide has shown better outcome compared to steroids alone. Most of the studies recommend treatment for one year [8].

## Conclusion

As PAN peaks around the age where atherosclerotic disease is also common, vasculitis should always be considered in the differential diagnosis of the patient presenting with ischemic gangrene and unexplained retroperitoneal bleeding.

## Consent

Written informed consent was obtained from the patient for publication of this case report and accompanying images. A copy of the written consent is available for review by the Editor-in-Chief of this journal.

## Competing interests

The authors declare that they have no competing interests.

## Authors' contributions

All the four authors contributed to the conception, design, analysis and preparation of the manuscript. All authors read and approved the final manuscript.

## References

[B1] Mahr A Guillevin L Poissonnet M Ayme S Prevalences of polyarteritis nodosa, microscopic polyangiitis, Wegener's granulomatosis, and Churg-Strauss syndrome in a French urban multiethnic population in 2000: a capture-recapture estimate Arthritis Rheum 2004 51 92 99 10.1002/art.20077 14872461

[B2] Watts RA Lane SE Bentham G Scott DG Epidemiology of systemic vasculitis: a ten-year study in the United Kingdom Arthritis Rheum 2000 43 414 419 10.1002/1529-0131(200002)43:2<414::AID-ANR23>3.0.CO;2-0 10693883

[B3] Ebert EC Hagspiel KD Nagar M Schlesinger N Gastrointestinal involvement in polyarteritis nodosa Clin Gastroenterol Hepatol 2008 6 960 966 10.1016/j.cgh.2008.04.004 18585977

[B4] Painter RW Sequential gastrointestinal complications of polyarteritis nodosa Am J Gastroenterol 1971 55 383 386 4397993

[B5] Ewald EA Griffin D McCune WJ Correlation of angiographic abnormalities with disease manifestations and disease severity in polyarteritis nodosa J Rheumatol 1987 14 952 956 2892931

[B6] Cornfield JZ Johnson ML Dolehide J Fowler JE Jr Massive renal hemorrhage owing to polyarteritis nodosa J Urol 1988 140 808 809 2901500 10.1016/s0022-5347(17)41821-0

[B7] Lightfoot RW Jr Michel BA Bloch DA Hunder GG Zvaifler NJ McShane DJ Arend WP Calabrese LH Leavitt RY Lie JT Masi AT Mills JA Stevens MB Wallace SL The American College of Rheumatology 1990 criteria for the classification of polyarteritis nodosa Arthritis Rheum 1990 33 1088 1093 10.1002/art.1780330805 1975174

[B8] Leib ES Restivo C Paulus HE Immunosuppressive and corticosteroid therapy of polyarteritis nodosa Am J Med 1979 67 941 947 10.1016/0002-9343(79)90634-X 42314

